# Supplementary material for: Complex‐centric proteome profiling by SEC‐SWATH‐MS
Source: Mol Syst Biol. 2019 Jan 14;15(1):e8438. doi: 10.15252/msb.20188438 (PMC6346213; doi:10.15252/msb.20188438)
Supplement: Supplementary file 7 — Dataset EV6 [file MSB-15-e8438-s007.zip › feature_plots_bioplex/Q15024.pdf]

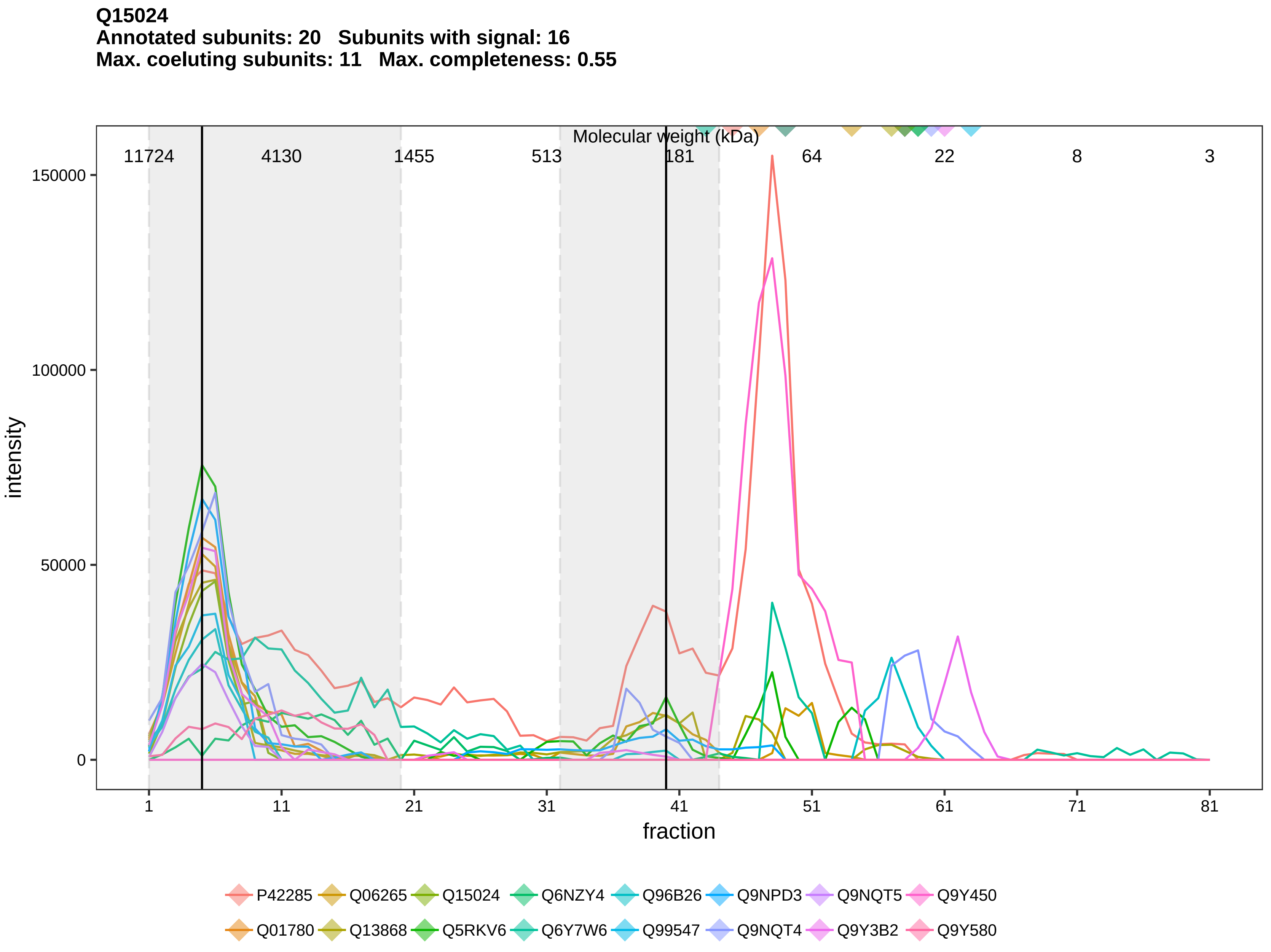
Q15024
Annotated subunits: 20 Subunits with signal: 16
Max. coeluting subunits: 11 Max. completeness: 0.55
Molecular weight (kDa)
11724
4130
1455
513
181
64
22
8
3
intensity
150000
100000
50000
0
fraction
1
11
21
31
41
51
61
71
81
P42285
Q06265
Q15024
Q6NZY4
Q96B26
Q9NPD3
Q9NQT5
Q9Y450
Q01780
Q13868
Q5RKV6
Q6Y7W6
Q99547
Q9NQT4
Q9Y3B2
Q9Y580
